# Impact of COVID on Professionals Addressing Psychosocial Needs of People With Diabetes: An International Survey

**DOI:** 10.3389/fcdhc.2022.828719

**Published:** 2022-03-17

**Authors:** Julie Wagner, Caroline Cummings, Richard Feinn, Karin Kanc, Miha Kos

**Affiliations:** ^1^Division of Behavioral Sciences and Community Health, School of Dental Medicine, University of Connecticut, Farmington, CT, United States; ^2^Department of Psychiatry, School of Medicine, University of Connecticut, Farmington, CT, United States; ^3^Department of Psychological Sciences, Texas Tech University, Lubbock, TX, United States; ^4^Department of Medical Sciences, Frank H. Netter School of Medicine, Quinnipiac University, Hamden, CT, United States; ^5^Jazindiabetes, Private Diabetes Centre, Ljubljana, Slovenia; ^6^Ustanova Hiša eksperimentov, Ljubljana, Slovenia

**Keywords:** diabetes, telemedicine, technology, COVID, social distancing, health professional, psychosocial, workforce

## Abstract

We investigated how COVID-19 has disrupted the work of health professionals who address behavioral and psychosocial needs of people with diabetes (PWD). English language emails were sent to members of five organizations that address psychosocial aspects of diabetes, inviting them to complete a one-time, anonymous, online survey. On a scale from 1=no problem, to 5=serious problem, respondents reported problems with the healthcare system, their workplaces, technology, and concerns about the PWD with whom they work. Respondents (n=123) were from 27 countries, primarily in Europe and North America. The typical respondent was a woman, aged 31-40 years, who worked in an urban hospital in medicine or psychology/psychotherapy. Most judged that the COVID lockdown in their region was moderate or severe. Over half felt moderate to serious levels of stress/burnout or mental health issues. Most participants reported moderate to severe problems due to the lack of clear public health guidelines, concerns with COVID safety of themselves, PWD, and staff, and a lack of access or knowledge on the part of PWD to use diabetes technology and telemedicine. In addition, most participants reported concerns with the psychosocial functioning of PWD during the pandemic. Overall, the pattern of findings reveals a high level of detrimental impact, some of which may be ameliorated with changes in policy and additional services for both health professionals and the PWD with whom they work. Concerns about PWD during the pandemic must go beyond their medical management and also consider the health professionals who provide them with behavioral and psychosocial support.

## Introduction

COVID-19 has disrupted the work of health professionals who work with people with diabetes (PWD) (1–3). The most common reasons for the disruption of health services are cancellations of scheduled treatments, implementation of lockdowns, community quarantines or movement control (4), and a lack of staff due to reassignment of a number of health professionals to COVID-19 activities (5). Risk of travel by public transportation and reluctance on the part of PWD to venture out for appointments is also a factor. Routine care is important not only for medical management, but also for providing diabetes self-management support and detection and treatment of diabetes distress and other psychosocial problems (6). Behavioral diabetes research has also been disrupted by COVID-19, causing delay and cancelations of study assessments and delivery of experimental behavioral interventions. Health professionals working with PWD have had to quickly adapt to these changes and learn ways to address behavioral and psychosocial needs of PWD and conduct behavioral diabetes research in the midst of the pandemic.

The toll of COVID-19 on frontline healthcare workers delivering lifesaving care to COVID-19 positive patients is well documented (7). Yet, the impact of COVID on the work of health professionals addressing the behavioral and psychosocial needs of PWD has not been investigated. This study was conducted to explore the nature and severity of those impacts. In an international group of health professionals who address behavioral and psychosocial needs of PWD, we explored problems that they have experienced with the healthcare system, their workplaces, technology, and concerns about the behavioral and psychosocial functioning of the PWD with whom they work.

## Methods

### Procedures

All procedures were approved by the UConn Health institutional review board (4/15/2021 IRB# 21X-219-1) which determined the research to be exempt. Data were collected over 6 months between April 2021 and October 2021. Participants provided online consent. Anonymous survey data were collected *via* Remote Electronic Data Capture [REDCap (8)].

### Sampling

English language emails were sent to invite participation in a one-time, anonymous, online survey. Invitations and a link to the survey were sent to five organizations whose members address psychosocial aspects of diabetes: 1) the psychosocial study group of the European Association for the Study of Diabetes (Psychosocial Aspects of Diabetes [PSAD, ~110 members]); 2) a behavioral diabetes research society (Behavioral Research in Diabetes Group Exchange [BRIDGE, ~125 members]); 3) the mental health interest group of the International Society for Pediatric and Adolescent Diabetes (ISPAD ~125 members); 4) the American Diabetes Association mental health care provider directory (~75 emails); 5) the diabetes special interest group of the Society of Behavioral Medicine (~200 members), and, 6) attendees at the 2021 Slovenia-based DiaMind online conference (~100 attendees), for a total of approximately 735 invitations. After providing online informed consent, participants responded to 50 questions. There were no incentives for participation.

### Measures

All survey questions were in English. Participants reported demographics, the characteristics of their workplace, the region in which they work, and the population of PWD with whom they work. Participants reported the country in which they work and these were coded according to World Bank gross national income (GNI) per capita categories as 1=low income ($1,045 or less), 2=lower middle income ($1,046 and $4,095), 3=upper middle income ($4,096 and $12,695), and 4=high income ($12,696 or more) (9).

For reporting problems, the instructions were as follows. “You may work with people with various medical conditions. We are interested in your work with people with diabetes. The people with diabetes that you work with may be your patients, clients, research participants, or some combination. Think about your own experience of working during the pandemic. Things may have changed over time with different surges, lockdowns, and re-openings. In general, over the past year, which of the following COVID-related issues has been a problem in your work with people with diabetes?” Response options were on the following scale: 1=no problem, 2=minor problem, 3=moderate problem, 4=somewhat serious problem, to 5=serious problem, “not applicable” and “prefer not to say”. We used this response option because it was applicable to our research question, and also because it is familiar to many behavioral diabetes health professionals from the Problem Area in Diabetes scale [PAID (10)].

Questions pertained to problems with the healthcare system, in their workplace, with technology, and concerns they had regarding the PWD with whom they worked. One question asked about the respondent’s level of stress/burnout/mental health issues. Items were generated by two of the authors (KK and JW) with qualitative input from colleagues based on a review of the literature and clinical experience.

### Data Analysis

Descriptive statistics included means with standard deviations for quantitative variables and frequencies with percentages for categorical variables. Data were analyzed using SPSS v27.

## Results

### Sample Characteristics

Of the 735 invitations, 149 people (20%) accessed the online consent. Of them, one recruit declined informed consent, 25 provided consent but did not complete the survey, while 123 consented and completed the survey, for a 17% completion rate. The majority of respondents were from Europe (45.5%), particularly Slovenia (19.5%), and North America (32.8%), predominantly the US (29.3%). Other countries included Australia, Austria, Belgium, Cambodia, Canada, Chile, Croatia, Ecuador, Egypt, Germany, India, Indonesia, Ireland, Italy, Jamaica, Mauritius, Mexico, the Netherlands, New Zealand, Romania, Russia, Saudi Arabia, Serbia, Slovakia, Turkey, and the United Kingdom. Most were from high (81.4%) and upper middle (11.5%) income countries. The typical respondent was a woman (74.8%), aged 31-40 years (38.2%), worked in an urban (77.2%) hospital (35.2%) and was from the discipline of medicine (47.5%) or psychology/psychotherapy (32.8%). About 1/3 (34.1%) used telemedicine prior to COVID and of those who did not, 80.5% adopted telemedicine due to COVID.

Most respondents (75.4%) worked with more than three others in their same profession and 46% saw between 51-500 PWD annually. About half saw only adult PWD and one-third worked exclusively with children. Most (90.2%) judged that the COVID lockdown in their region was moderate or severe. In response to a single question, over half (56.4%) reported moderate to serious levels of stress/burnout/mental health issues. Only 1% reported that COVID has not been a problem with their work with PWD. See **Table 1** for descriptives.

**Table 1:** Demographic characteristics of respondents (n=123).

Characteristic	Frequency	Percentage
Gender		
Female	92	74.8%
Male	31	25.2
Age Group		
<30	6	4.9%
31-40	47	38.2
41-50	27	22.0
51-60	18	14.6
61-70	14	11.4
>70	11	8.9
Country Region		
Africa/Middle East	6	5.2%
Asia/Oceania	9	7.8
Europe	56	45.5
United Kingdom	3	2.4
North America	38	32.8
South America/Caribbean	4	3.3
Country Income Level		
Low	0	0.0%
Low Middle	8	7.0
High Middle	13	11.5
High	92	81.4
Urbanicity		
Urban	95	77.2%
Suburban	19	15.4
Rural	9	7.3
Severity of COVID Lockdown		
Mild	11	8.9%
Moderate	57	46.3
Severe	54	43.9
Personal COVID-Related Stress/Burnout/Mental Health		
Not a Problem	11	10.7%
Minor Problem	34	33.0
Moderate Problem	35	34.0
Somewhat Serious Problem	15	14.6
Serious Problem	8	7.8
Work Setting		
Primary Care	2	1.6%
Community Health Center	5	4.1
Specialty Care	22	18.0
Academic	28	23.0
Hospital	43	35.2
Private Practice	17	13.9
Other	5	4.1
Discipline		
Medicine	58	47.5%
Nursing	10	8.2
Psychology/Psychotherapy	40	32.8
Social Work	2	1.6
Nutrition	4	3.3
Other	8	6.6
Primary Professional Activity		
Medical Care	56	45.5%
Mental Health Care	27	22.0
Diabetes Education	13	10.6
Clinical Research	23	18.7
Other	4	3.3
Number of Employees in Your Profession in your Workplace		
I’m the only one	10	8.2%
2-3	20	16.4
>3	92	75.4
PWD Seen in a Year		
<50	24	19.5%
51-500	57	46.3
510-1000	21	17.1
>1000	21	17.1
Type of Diabetes of PWD		
Type 1	47	38.5%
Type 2	28	23.0
Mixed	47	38.5
Age of PWD		
Pediatric	41	33.3%
Adult	61	49.6
Mixed	21	17.1
Socioeconomic Status of PWD		
Low	11	8.9%
Medium	30	24.4
High	1	0.8
Mixed	81	65.9
How Much COVID affected Your Work with PWD		
Not a Problem	1	1.0%
Minor Problem	28	28.0
Moderate Problem	40	40.0
Somewhat Serious Problem	21	21.0
Serious Problem	10	10.0

PWD, People with diabetes, i.e., people with whom the respondent works.

### Problems With the Healthcare System

Below we provide the percentage of respondents reporting a problem as moderate to serious. As seen in **Table 2**, with respect to the healthcare system the greatest problem reported was lack of clear public health guidelines, where 56.4% reported it was a moderate to serious problem. Other common problems were delay or lack of access to COVID testing (32.0%) and COVID-related issues with reimbursement for their work (30.7%). Over 1/5 of respondents reported disagreement about COVID-related work issues with government bodies (28.3%), their organization’s administration (23.8%), and research funders (22.6%).

**Table 2:** Problems with the healthcare system (Scale Range 1= not a problem to 5 = serious problem).

Problem	Mean	SD	Percentage Moderate to Serious
Lack of public health guidelines for COVID	2.77	1.30	56.4%
Delay/lack of access to COVID testing for peoples with diabetes	2.11	1.12	32.0%
COVID related issues regarding reimbursement	1.93	1.08	30.7%
COVID related issues regarding documentation	1.88	0.99	26.3%
Delay/lack of access to COVID testing for you and staff	1.69	1.13	18.4%
Disagreement about work-related COVID issues with:			
Government bodies	1.96	1.27	28.3%
Organization administration	1.84	1.02	23.8%
Research funders	1.80	1.28	22.6%
Licensure organizations	1.59	1.16	16.0%
Human subjects protection and regulatory bodies	1.59	1.02	18.6%
Colleagues	1.59	0.91	14.7%

### Problems in the Workplace

Workplace problems were abundant (**Table 3**). Over half the respondents reported moderate to severe problems regarding COVID safety at work, including COVID safety for PWD (69.0%), changing the flow of people in the workspace (69.3%), safety of self and staff (62.4%), making decisions to close or reopen (59.3%), and possibly exposing family members to COVID from a work-related exposure (50.9%). Nearly half (48.5%) reported isolation from colleagues as a moderate to serious problem.

**Table 3:** Problems in the workplace (Scale Range 1= not a problem to 5 = serious problem).

Problem	Mean	SD	Percentage Moderate to Serious
COVID safety for PWD	3.12	1.18	69.0%
Changing flow of people through workspace	3.06	1.16	69.3%
Your own and your staff COVID safety	2.85	1.16	62.4%
Making decision to close/reopen workplace	2.81	1.32	59.3%
Possibly exposing family to COVID	2.68	1.24	50.9%
Isolation from colleagues	2.50	1.14	48.5%
Disruption to relationship between you and PWD	2.42	1.15	44.3%
Skepticism about COVID among PWD	2.34	1.10	40.0%
Financial consequences of COVID to work	2.27	1.30	42.1%
Lack of time with PWD	2.20	1.19	34.7%
Uncertainty how to explain COVID risks to PWD	2.09	1.11	32.6%
Wearing personal protection equipment	2.06	1.08	30.7%
Disruption of supply chains	2.06	1.09	29.5%
Keeping up with COVID research	1.97	1.04	32.7%
Needing to quarantine from family due to possible exposure at work	1.93	1.17	28.3%
Staff vaccine hesitancy	1.88	1.09	25.0%
Skepticism about COVID among your staff	1.72	0.94	17.9%
Your own vaccine hesitancy	1.11	0.50	2.1%

PWD, People with diabetes, i.e., people with whom the respondent works.

Working with PWD was impacted; 44.3% reported that disruption to the relationship with PWD was a moderate to serious problem and over 1/3 (34.7%) reported lack of time with PWD. Skepticism about COVID among PWD was reported by 40%, and uncertainty how to explain COVID risks by 1/3 (32.6%).

### Problems With Technology

One set of technology questions pertained to diabetes technology (CGMs, uploadable pumps, data sharing platforms). The majority of respondents reported that “PWD not having access or knowledge to use diabetes technology” was a moderate to serious problem (54.5%; mean = 2.7, SD = 1.1) but that their own use of diabetes technology was less problematic (22.1%; mean = 1.8, SD = 0.9).

Another set of technology questions pertained to telemedicine. The most frequently reported moderate to serious technology problems included “PWD not having access or knowledge of telemedicine” (59.1%; mean = 2.9, SD = 1.0), “too much screen time from telemedicine” (46.7%; mean = 2.6, SD = 1.2), and “legal, licensure, reimbursement issues with telemedicine” (35.3%; mean = 2.2, SD = 1.3). Other issues included “Needing to learn to use telemedicine” (17.3%; mean = 1.8, SD = 1.0) and “not having access to telemedicine (21.5%; mean = 1.74, SD = 1.1).

### Concerns About PWD

**Figure 1:**
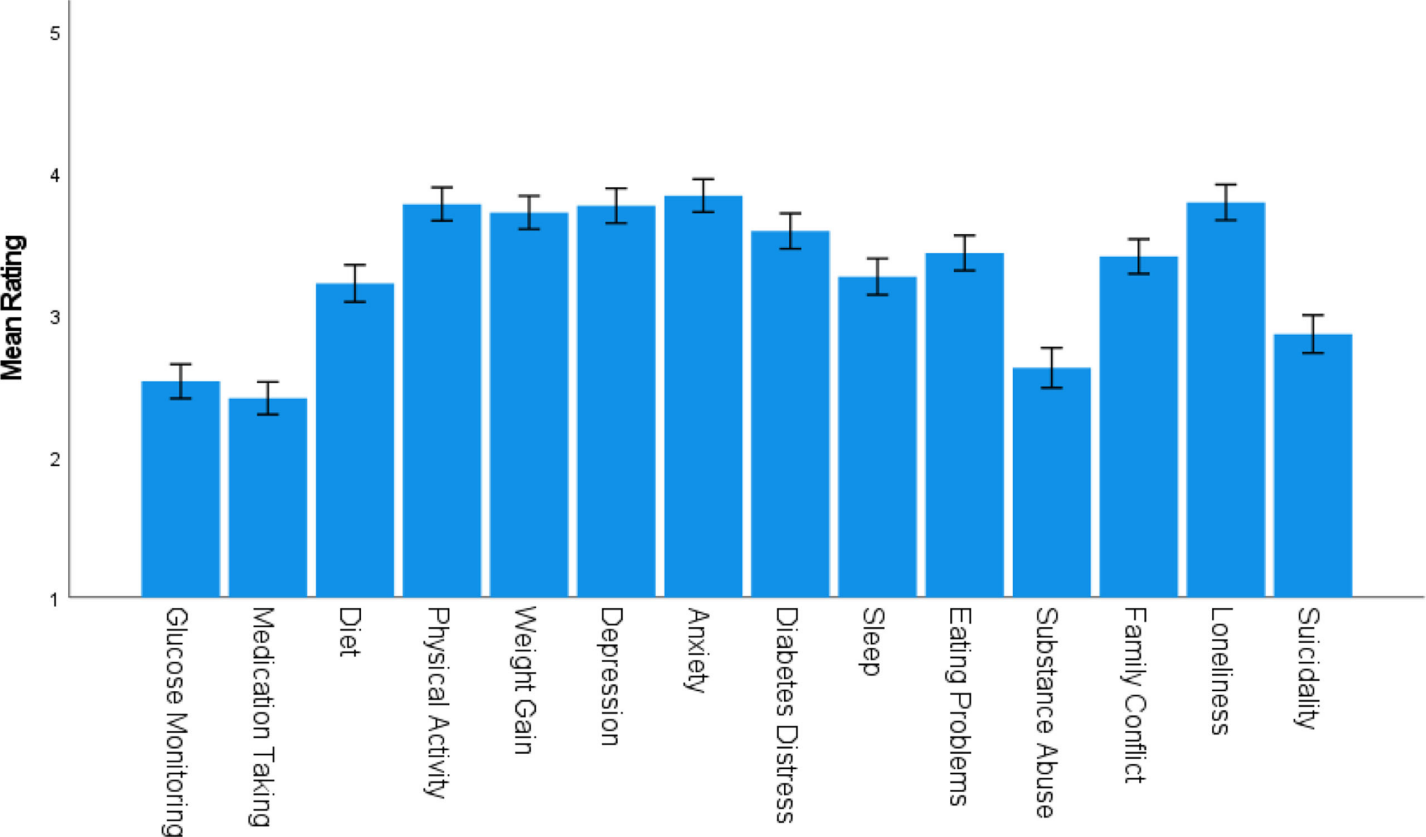
Mean (± SEM) Concerns Regarding PWD. PWD, People with diabetes, i.e., people with whom the respondent works.

**Figure 1** shows the mean scale scores to questions regarding respondent concerns about the impact of COVID on the behavioral and psychosocial wellbeing of PWD; most means are above three (moderate problem). Behaviorally, the top concerns were physical activity (90%), weight gain (89%), eating problems (79%), diet (75%), and sleep (72%). Regarding psychosocial wellbeing, top concerns were anxiety (90%), loneliness (87%), depression and diabetes distress (both 83%), and family conflict (74%). Suicidality among PWD was reported as a moderate to serious problem by 52% of respondents.

## Discussion

The current manuscript describes the impact of the COVID-19 pandemic as reported by professionals working to address the psychosocial needs of PWD. Most participants reported that they were experiencing moderate to serious levels of stress, burnout or mental health issues. They also reported problems due to the lack of clear public health guidelines, concerns with COVID safety for themselves, PWD, and staff. Another clear problem was PWD lacking access to, or not knowing how to use, diabetes technology and telemedicine. In addition, most participants reported concerns with the behavioral and psychosocial functioning of PWD during the pandemic and a disruption to the PWD relationship. Overall, the pattern of findings reveals a high level of detrimental impact, some of which may be ameliorated with changes in policy and additional services for both health professionals and the PWD with whom they work, at the levels of the healthcare system, institution, and workplace. Concerns about the health and wellbeing of the PWD during the pandemic must go beyond their medical management and also consider their need for behavioral and psychosocial support and the impact of COVID-19 on the health professionals who provide it.

First, our findings point to the risk of a potential decline in the already under-resourced workforce of professionals who address the behavioral and psychosocial needs of PWD. Both pediatric and adult treatment guidelines recommend psychosocial screening, intervention, or referral to mental health care when warranted (11). Yet, most settings do not offer this type or level of service. Whereas reimbursement continues to be a challenge, a shortage of professionals to meet the practice and research needs for PWD diabetes is a crucial factor. Over half of respondents reported that their own level of stress, burnout, and mental health was a moderate to serious problem. Healthcare professionals who are more psychologically impacted by the COVID-19 pandemic report greater intentions to quit or retire (12). Ignoring the needs of professionals who address psychosocial issues in PWD may result in a loss of workforce that is already seriously underpowered (13). This may be especially true for women health professionals, who report barriers to remaining in their career field, including work-family conflicts and workplace harassment (14). Indeed, in the context of the current pandemic, female health professionals report greater professional and family-related stress than their male counterparts (15). If the workforce declines, combined with limited support for workers—especially women—there will be downstream negative consequences for PWD, including a net loss in evidence-based intervention development and the delivery of clinical services to PWD exactly when these are needed most.

Second, many respondents reported significant problems with the healthcare system, their workplace, and access to technologies that would allow them to work safely and efficiently during COVID, providing specific areas for improvement of workplace functioning. Respondents were concerned about potential COVID exposure of themselves and in turn their families as well as exposure of their staff and PWD. Safety concerns were compounded by the limited patient access to telemedicine, precluding professionals from shifting to remote delivery of services which could have reduced exposure to workplace hazards. There are data suggesting that greater perceptions of exposure to workplace hazards may reduce the quality of life of healthcare professionals (16) and reduced quality of life of professionals is associated with reduced quality of care (17). Therefore, the continued stress associated with the pandemic may not only impact workplace functions, but also the well-being of professionals and the care PWD receive. It is imperative to identify ways to better support professionals working with PWD, which should prioritize elevating staff voices in the implementation of protocols.

Third, respondents reported significant concerns with both access to care and behavioral and psychosocial functioning of PWD, which highlights the importance of overcoming the aforementioned workplace hazards and obstacles to providing quality care to PWD. For example, respondents reported a perceived disruption to their relationship with PWD. There are extensive data highlighting the importance of the patient-provider relationship in predicting diabetes self-management in patients (18, 19). The disruption to this key relationship may in part explain the perceived reduction of patients’ engagement in key health behaviors (i.e., physical activity, diet, sleep) required for effective diabetes management. Respondent concerns are consistent with recent studies which indicate COVID-related disruptions in physical activity (20, 21), diet (20–23), sleep (20, 24) and diabetes self-care (25), among PWD. Disruption of the professional-provider relationship may also in part explain perceptions of the overall decrease in emotional wellbeing of patients (i.e., increased depression, anxiety, suicidality, diabetes distress, loneliness, and family conflict). Here, too, respondent concerns are consistent with the literature showing reduced psychosocial wellbeing (26) in PWD during the COVID-19 pandemic. Non-pharmacological interventions exist for many of the behavioral (27, 28) and psychosocial (29–31) problems faced by PWD. Unfortunately, respondents within our study are also the professionals specifically trained to screen, assess, and intervene on problems with diabetes self-management and wellbeing of PWD. Yet, due to the workplace hazards and other obstacles previously mentioned, these professionals are limited in the quality and quantity of services that can be provided. The pandemic is likely to continue (32) and climate disruption will likely bring natural disasters and associated social unrest that could also interrupt routine medical care for many. Initiating modified in-person diabetes services, and/or telemedicine-delivered services, including and especially psychological services to improve psychosocial wellbeing of PWD, is crucial to face these future challenges.

A lack of clear public guidelines was a top problem among participants. Lack of clear guidelines may be related to the disagreement that participants reported with various institutions such as government bodies, research funders, licensure organizations and human subjects protections committees. This is consistent with data highlighting inconsistencies in guidelines across various global and national organizations (33). It is likely also related to participants’ relatively low confidence in explaining COVID risk to PWD. Uncertainty about COVID-19 guidelines and vaccination has been studied across various health professions (34, 35) and is problematic because professionals may inadvertently transfer their uncertainty onto patients, thereby reinforcing distrust of the medical system. Moreover, data supports that knowledge about the COVID-19 vaccine, including its benefits, strongly predicts intention to accept the vaccine (36). Thus healthcare professionals play a key role in countries achieving herd immunity primarily by vaccination.

### Limitations

There are limitations to consider in the context of study findings. First, the response rate was low (20%) and the sample was small which limits the analyses that can be conducted and the generalizability of findings. Larger samples would allow comparison across respondents by discipline (e.g., medicine vs nursing vs. psychology), primary professional activity (e.g., clinical care vs research) and patient population (e.g., adults vs pediatrics). Of those who responded, most were middle-aged women from European countries and the United States; thus, self-selection bias may have impacted results. Of note, the majority of the sample was from Europe where women outnumber men in the health professions (37) thus, our preponderance of women may not be unrepresentative. Future research should include more respondents from low- and low-middle income countries where experiences of the pandemic may be very different from those reported here. Respondents were limited to English speakers and findings may not generalize to professionals who do not speak English. Second, study methodology required respondents to retrospectively report problems which can be influenced by forgetting and/or recall bias. The role of threat perception on responding should also be considered, as those who are more distressed may report more problems. Future research should also clearly define terms such as stress/burnout/mental health issues. Finally, data collection occurred during middle to late 2021, after the initial nationwide closures and re-openings occurred. Findings may not be representative of changes at other periods of the pandemic.

### Conclusion and Future Directions

The current study highlights professional perspectives of the effect of the COVID-19 pandemic on the healthcare system, workplace, technology, and behavioral and psychosocial functioning of PWD. Many of the reported concerns could be addressed through increasing public and professional knowledge about COVID-19 in a clear and timely manner. Organizations should attempt to provide trainings about how professionals can disseminate evidence-based knowledge about COVID-19 to PWD. This might include reference to emerging studies about the impact of COVID-19 social distancing measures on lifestyle and mental health of PWD. It could also include reference to studies about the impact of COVID-19 infection (38) and vaccine administration (39) on diabetes. More broadly, professional organizations should develop and disseminate trainings on providing care in the context of a global crisis.

In addition, given participants’ notable concerns with COVID-19 exposure, it will be important to ensure organizations are equipped with the technology needed to deliver services remotely. Organizations might consider reallocating funds to make telemedicine accessible for PWD and professionals, and providing trainings about how PWD and professionals can use telemedicine and troubleshoot any technology problems (e.g., camera angle). Data show that telemedicine, when properly introduced, is well received by PWD (40, 41) and providers (42). Importantly, consideration of a hybrid model of service delivery (i.e., remote and in-person) should be considered, even after the pandemic resolves. From a policy standpoint, it will also be important for insurance companies and licensing boards to allow for flexible provision of services across regions (e.g., across state lines in the United States) and across modalities (i.e., in-person and *via* telemedicine) over time and waves of the pandemic. Moreover, governments should prioritize providing equitable access to: 1) internet for all citizens to reduce barriers to telemedicine, 2) diabetes technologies to reduce the burden of daily diabetes management, 3) diabetes management supplies across all regions of the world, including building better infrastructure to protect supply chains, and, 4) personal protective equipment for all private and public healthcare settings to reduce exposure to workplace hazards.

Also, with the specter of a declining workforce, there is a need to increase the pipeline of professionals trained to address the biopsychosocial needs of PWD. This might include training bachelor’s or master’s level clinicians to provide supportive services or increasing the workforce of peer counselors and community health workers who can provide diabetes self-management education and support. Prior clinical trials support the efficacy and acceptability of interventions led by peer counselors (43, 44) and community health workers (45, 46). Therefore, such interventions should be delivered more broadly, especially in the context of the COVID-19 pandemic during which health and wellbeing of PWD are found to be impacted. Also, there are pilot data supporting the implementation of the Look AHEAD and Diabetes Prevention Program Group Lifestyle Balance program in providing support during the pandemic (47), thus the program should be implemented more widely. There may also be a role for mobile mental health applications to equip patients with readily accessible psychological support.

Professionals should also seek psychological care for themselves, as needed, to improve their own wellbeing, which is likely to have downstream effects on the care they provide (17). For example, mindfulness-based stress reduction interventions have been demonstrated to yield significant improvements in quality of life (48), anxiety, depression and stress (49) in healthcare professionals.

Last, and importantly, as hospitals, clinics and other diabetes service organizations continue to re-instate services, professionals might consider ways to re-establish the PWD-professional relationship. In a recent study, patients who were reached by phone were more than twice as likely to book and keep an appointment compared to those who were not reached or who had a voicemail message left (50). Therefore, providers might consider making direct contact with patients to increase the likelihood that patients will re-initiate access to care, as well as to provide an opportunity for a positive, brief interaction between the patient and their provider, thereby taking the first step towards rebuilding this important relationship.

## Data Availability Statement

The raw data supporting the conclusions of this article will be made available by the authors, without undue reservation.

## Ethics Statement

The studies involving human participants were reviewed and approved by UConn Health IRB. The participants electronically provided their signed informed consent to participate in this study.

## Author Contributions

JW, KK, and MK conceived and implemented the study. RF conducted data analysis. CC contributed to writing the manuscript. All authors read and edited the manuscript. All authors contributed to the article and approved the submitted version.

## Conflict of Interest

The authors declare that the research was conducted in the absence of any commercial or financial relationships that could be construed as a potential conflict of interest.

## Publisher’s Note

All claims expressed in this article are solely those of the authors and do not necessarily represent those of their affiliated organizations, or those of the publisher, the editors and the reviewers. Any product that may be evaluated in this article, or claim that may be made by its manufacturer, is not guaranteed or endorsed by the publisher.
